# Effectiveness of self-empowerment-affirmation-relaxation (Self-EAR) program for postpartum blues mothers: A randomize controlled trial

**DOI:** 10.12669/pjms.346.15986

**Published:** 2018

**Authors:** Krittipitch Thitipitchayanant, Ratana Somrongthong, Ramesh Kumar, Naowarat Kanchanakharn

**Affiliations:** 1 *Krittipitch Thitipitchayanant, School of Nursing, University of Phayao, Thailand. College of Public Health Sciences, Chulalongkorn University, Thailand*; 2 *Ratana Somrongthong, College of Public Health Sciences, Chulalongkorn University, Thailand*; 3 *Ramesh Kumar, Health Services Academy Islamabad, Pakistan*; 4 *Naowarat Kanchanakharn, College of Public Health Sciences, Chulalongkorn University, Thailand*

**Keywords:** Postpartum blues, Self-EAR program, Self-Empowerment, Self-Affirmation, Progressive muscle relaxation, Newly blues mothers

## Abstract

**Background and Objecvites:**

Approximately 55-85% of women worldwide have experienced postpartum blues (PPB) during 6-9 weeks after delivery without receiving the counseling program; more than 20% of them have developed into postpartum depression. Study objectives were to evaluate the effect of the Self-EAR program to improve the postpartum blues scores and serum allopregnanolone level among newly blues mothers.

**Methods:**

During June 2015 to May 2016, the randomized controlled trial was conducted among 76 Nulliparous blues mothers who were screened with Stein’s postpartum blues scores ≥ 3. All participants were randomly assigned either to the intervention group (Self-EAR program) and the control group (standard postpartum care program). The Self-EAR program was transformed into audio files which were installed in an MP3 digital device before providing it to the intervention group in order to be implemented at home three times per day for four weeks. Participants were assessed at baseline, 1-month, 2-month and 3-month follow-up for serum allopregnanolone level. Data were analyzed by using descriptive statistic, chi-square test, t-test, and repeated measure analysis of variance.

**Result:**

After the 3-month follow-up, the results revealed positive effects of the Self-EAR program on postpartum blues scores (p-value=0.002) and serum allopregnanolone concertation (p-value=0.001). The participants in the intervention group had experienced significantly lower postpartum blues scores; on the other hand, they had significantly higher serum allopregnanolone level when compared with the control group.

**Conclusions:**

The findings suggested that the Self-EAR program was effective to improve postpartum blues scores and allopregnanolone level among newly postpartum blues mothers.

## INTRODUCTION

Usually after childbirth, most women experienced face fluctuated emotion or mood swings, including teary eyes, Irritability, sleep disturbance, lack of concentration, and absent-minded within the first week until 6-9 weeks postpartum.^1^ All conditions are called “Postpartum Blues (PPB)” or “Maternity Blues” or “Baby Blues”; and its incidence rate is estimated at 55-85% in western country.^2^ PPB was classified as a mental health adaptation condition that can arouse negative effects to physical and mental health of mothers and infants. Without receiving the guiding and caring program that helps women to cope with their condition, more than 20% can develop the postpartum depression (PPD). On the other hand, if women are given the physical and psychological counseling program properly, these symptoms will disappear in a few days without using psychiatric drug.^3^ The reviewers of Neuro-Psychopharmacology & Biological Psychiatry have identified an involvement of Allopregnanolone (AP) neurosteroids or neuroactive steroids level in both modulating and detecting stress and stress-related disorders including anxiety, panic, and depression.^4^-^6^

In the physical change of PPB, many studies of hormone during postpartum periods were contradicting reports, for example, progesterone, estradiol, and cortisol.^7^-^9^ Only allopregnanolone played an important role as anxiolytic, hypnotic, and anticonvulsant effects regulated the pathophysiology of emotional disorders including depression, anxiety, and stress related disorders.^10^-^12^ In the psychological change aspects, many experimental studies investigated the effectiveness of the program to improve mental health in both pregnant women and non-pregnant women including self-empowerment^13^, self-affirmation^14^,^15^ and relaxation program.^16^ Only one aspect in a program cannot handle this situation. Whereupon this study created the Self-Empowerment-Affirmation-Relaxation [Self-EAR] program, installed in an alternative audio MP3 digital files, and designed for newly blues mothers, with the aim to evaluate the effectiveness of the Self-EAR program on improving of the postpartum blues scores and serum allopregnanolone levels.

## METHODS

A randomized controlled trial was conducted in rural Thailand from June 2015 to May 2016. Participants were given an adequate verbal explanation of the trial; they were asked to sign written informed consent for participation. The study protocol was approved by the Ethics Review Committee for Research Involving Human Research Subjects, Health Science Group, Chulalongkorn University (no.122/2015). The inclusion criteria were as follows, willingness, nulliparous mothers who were screened by Stein’s postpartum blues questionnaire ≥ 3 and screened by Edinburgh Perinatal Depression Scale (EPDS) < 13; maternal aged 20 to 35 years. Those who had complications from medical and obstetrical complication; psychosis disorder; on antipsychotic medication, unable to understand and read Thai; and unaltered accommodation after three months of childbirth were excluded. Participants were randomly assigned to the intervention and the control groups by using simple random sampling (SRS). The mean delivery conducted in hospital was 1,753 cases per year. Around 80 participants were eligible from 150 women were admitted to the postpartum unit. Hence, 40 women were allocated in each group by using SRS. There was one dropout participant in the intervention group and three dropout participants in the control group with the reason of moving to another province. The team was trained by the expert in laboratories to operate Allopregnanolone ELISA kits. Stein’s postpartum blues questionnaire^1^ (Q1) was used for postpartum blues scores (Cronbach alpha = 0.780), EPDS questionnaire^17^ (Q2) was used to ensure participants that they do not have postpartum depression at the beginning of the study (Cronbach alpha = 0.825) and Human allopregnanolone ELISA kits were used to measure serum allopregnanolone concentration. Eighty percent of power calculations were performed based on a previous study24 of the 5% significance level; and increase 20% in the dropout of participants was added for both groups. Descriptive statistics, chi-square, and t-tests were used to compare the differences between the intervention and the control groups at baseline. Repeated-measures ANOVA was used to compare the change in outcomes across time.

### Intervention

The Self-EAR program is based on the self-empowerment, the self-affirmation, and relaxation techniques. The Self-Empowerment techniques proceeded the three aspects approach including Self-Control, Self-Motivation, and Self-reinforcement. The Self-Affirmation techniques used to guide behavior and decisions, especially to cope with a negative thinking by repeating affirmations to oneself every day and every time. The relaxation techniques practiced by using the progressive muscle relaxation. In the pre-research phase, a focus group discussion was used to develop the 10-minute MP3 audio files by brainstorming ideas from 11 postpartum blues mothers and four nursing instructors. The Self-EAR program was transformed into audio files which was installed in an MP3 digital device before providing it to the intervention group to be implemented at home three times per day for 4 weeks. The control group received the regular and routine standard postpartum care program.^18^ Participants in both groups answered self-report questionnaires of postpartum blues at baseline, 1-month, 2-month and 3-month follow up.

## RESULTS

The demographic characteristics of participants at baseline are shown in Table-I. Among the 76 participants with PPB (39 postpartum mothers in the intervention group and 37 postpartum mothers in the control group), most of them were Buddhist and graduated secondary education. The average age of the participants in the intervention and the control groups were 23.69±3.79 and 23.78±4.33 years, respectively. There were no statistically significant differences in age, education level, occupation, religions, gestational age, and type of delivery between the intervention and control groups (p=0.804, 0.795, 0.535, 0.610, 0.175, and 0.491 respectively) (Table-I).

**Table-I:** Demographic characteristics of theresponents.

Characteristics	Intervention n (%)	Control n (%)	p-value
***Age (years)***			
Mean (SD)	23.69 (3.79)	23.78 (4.33)	0.804
***Educational level***			
Primary education	1 (2.60)	2 (5.40)	0.795
Secondary education	23 (59.0)	24 (64.90)	
Diploma	5 (12.80)	3 (8.10)	
Bachelor	10 (25.60)	8 (21.60)	
***Occupation***			
No careers	18 (46.20)	13 (35.10)	0.535
Employees	13 (33.30)	15 (40.50)	
Merchants	7 (17.90)	5 (13.50)	
Farmers	1 (2.60)	4 (10.80)	
***Religion***			
Buddhist	38 (97.40)	35 (94.60)	0.610
Christian	1 (2.60)	2 (5.40)	

### Comparison of Mean

**Fig.1:**
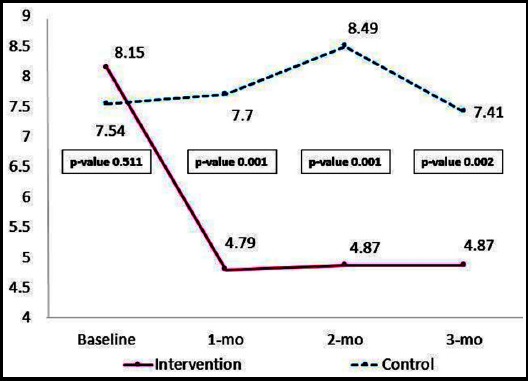
Comparison of Postpartum Blues Scores after implementing the Self-EAR program at Baseline and 3 times follow up.

Comparison of mean postpartum blues scores presented in Fig.1. The mean of postpartum blues scores had decreased significantly between the intervention and the control group at 1-month, 2-month, and 3-month follow-up (p-value 0.001, 0.001, and 0.002 respectively).

**Fig.2:**
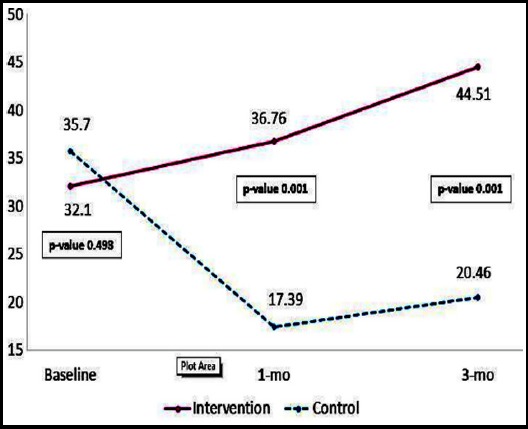
Comparison of Allopregnanolone level after implementing the Self-EAR program at Baseline and 2 times follow up.

Comparison of mean allopregnanolone serum level is presented in Fig.2. The mean of allopregnanolone serum level had increased significantly between the intervention and the control group at 1-month, and 3-month follow-up (p-value 0.001, and 0.001 respectively).

### Repeated-measures ANOVA

The participants in the intervention group had a significant difference in postpartum blues scores (p=<0.05) and serum allopregnanolone level (p=<0.05) when compared with the control group. Findings showed only postpartum blues scores had statistically significant differences in both group measurements and the interaction effect between measurements depending on groups, whereas serum allopregnanolone level yielded statistically significant differences only in the interaction effect between measurements depending on groups (p=0.001) (Table-II).

**Table-II:** Repeated Measures ANOVA of postpartum blues scores and Allopregnanolone serum level.

Postpartum Blues Scores	Source	SS	df	MS	F	p-value
Between subject	Intervention	84.588	1	84.588	9.89	0.002*
	Error	632.915	74	8.553		
Within subject	Time	139.137	2.347	59.275	5.098	0.005*
	Intervention x Time	199.295	2.347	84.903	7.302	0.001*
	Error	2019.577	173.702	11.627		
Allopregnanolone serum level						
Between subject	Intervention	10035.024	1	10035.024	20.368	0.001*
	Error	36458.073	74	492.677		
Within subject	Time	1970.218	2	985.109	2.607	0.077
	Intervention x Time	8317.260	2	4158.63	11.007	0.001*
	Error	55915.327	148	377.806		

### Pairwise comparisons of different time measurements

The mean difference of postpartum blues scores between the intervention and the control groups was lowest at 1-month, 2-month, and 3-month follow up (p=0.001, 0.001, and 0.002, respectively). The mean difference of serum allopregnanolone level between the intervention and control groups was highest at 1-month and 3-month follow up (p=0.001, and 0.001, respectively). (Table-III).

**Table-III:** Pairwise Comparisons of Different Time Measurements of Blues scores and Allopregnanolone level in Intervention and Control Groups

Time of data collection	Mean Difference	SE	95% CI		p-value
			Lower	Upper
***Blues scores***					
Baseline	0.613	0.929	-1.238	2.464	0.511
1 month follow up	-2.908	0.775	-4.452	-1.363	0.001*
2 month follow up	-3.615	1.068	-5.742	-1.487	0.001*
3 month follow up	-2.534	0.797	-4.122	-0.945	0.002*
***Allopregnanolone level***					
Baseline	-3.6018	5.2274	-14.0176	6.8139	0.493
1 month follow up	19.3750	4.3900	10.6278	28.1222	0.001*
3 month follow up	24.0461	4.3758	15.3270	32.7650	0.001*

## DISCUSSION

This research study demonstrates the effectiveness of the Self-EAR program that integrated three techniques uniquely to new mothers who had faced with the blues after childbirth. Both postpartum blues scores and allopregnanolone serum level in the newly blues mothers were improved and sustained to 3-month follow-up. The Self-EAR program decreased postpartum blues scores by increasing allopregnanolone serum level. Previous researchers, who conducted progressive muscle relaxation among pregnant women, discovered an improvement in well-being outcomes such as reduced pain level, perceived stress, and promote quality of life.^4^,^5^,^19^,^20^ Furthermore, they discovered that the progressive muscle relaxation (PMR) program significantly sustained improvement on depression, anxiety, and quality of life among chronic patients.^13^,^21^-^23^ The results from a review and meta-analysis publication illustrated that stress reduction program are able to reduce stress level in healthy people.^24^-^26^ Guardino et.al.^27^ and Jallo et.al.^28^, both of them conducted the RCT with stress controlling programs for pregnant women with high level of stress; and they found that the interventions may effectively reduced anxiety and may have potential stress coping benefits. However, the postpartum depression is common problem in Thailand reported by the recent survey.^29^ Nevertheless, no studies conducted a specific intervention to reduce blues during postpartum periods. The Self-EAR program, including three techniques: self-empowerment, self-affirmation, and progressive muscle relaxation, which covered all of the aspects for postpartum blues mothers. The program improved both postpartum blues scores and allopregnanolone serum level and sustained up to the 3-month follow up. The strengths of this current research were as follows: 1) used self-empowerment technique to control their emotion 2) used self-affirmation technique to practice every day and 3) used relaxation technique to relax their body stress; therefore the Self-EAR program covered all of the aspects for postpartum blues mothers and sustained up to the 3-month follow up. The research Limitations of the study: Willingness of the participants which could create the selection bias. The Self-EAR program has the effectiveness and acceptability that can be adapted into daily practice at home for all populations. Furthermore this study can be implemented among larger population for a longer follow up time.

## CONCLUSION

The findings suggested that the Self-EAR program can be applied to decrease postpartum blues scores and increase allopregnanolone level among newly postpartum blues mothers.
